# Investigation of the Impact of *CYP3A5* Polymorphism on Drug–Drug Interaction between Tacrolimus and Schisantherin A/Schisandrin A Based on Physiologically-Based Pharmacokinetic Modeling

**DOI:** 10.3390/ph14030198

**Published:** 2021-02-27

**Authors:** Qingfeng He, Fengjiao Bu, Hongyan Zhang, Qizhen Wang, Zhijia Tang, Jing Yuan, Hai-Shu Lin, Xiaoqiang Xiang

**Affiliations:** 1 Department of Clinical Pharmacy and Pharmacy Administration, School of Pharmacy, Fudan University, Shanghai 201203, China; qf_he@fudan.edu.cn (Q.H.); 13211030039@fudan.edu.cn (F.B.); 14211030045@fudan.edu.cn (H.Z.); 16211030062@fudan.edu.cn (Q.W.); zjtang@fudan.edu.cn (Z.T.); jyuan@fudan.edu.cn (J.Y.); 2 College of Pharmacy, Shenzhen Technology University, Shenzhen 518118, China; linhaishu@sztu.edu.cn

**Keywords:** physiologically-based pharmacokinetic (PBPK), Wuzhi capsule (WZC), tacrolimus, *CYP3A5* polymorphism, drug–drug interaction (DDI), schisantherin A (STA), schisandrin A (SIA)

## Abstract

Wuzhi capsule (WZC) is commonly prescribed with tacrolimus in China to ease drug-induced hepatotoxicity. Two abundant active ingredients, schisantherin A (STA) and schisandrin A (SIA) are known to inhibit CYP3A enzymes and increase tacrolimus’s exposure. Our previous study has quantitatively demonstrated the contribution of STA and SIA to tacrolimus pharmacokinetics based on physiologically-based pharmacokinetic (PBPK) modeling. In the current work, we performed reversible inhibition (RI) and time-dependent inhibition (TDI) assays with *CYP3A5* genotyped human liver microsomes (HLMs), and further integrated the acquired parameters into the PBPK model to predict the drug–drug interaction (DDI) in patients with different *CYP3A5* alleles. The results indicated STA was a time-dependent and reversible inhibitor of CYP3A4 while only a reversible inhibitor of CYP3A5; SIA inhibited CYP3A4 and 3A5 in a time-dependent manner but also reversibly inhibited CYP3A5. The predicted fold-increases of tacrolimus exposure were 2.70 and 2.41, respectively, after the multidose simulations of STA. SIA also increased tacrolimus’s exposure but to a smaller extent compared to STA. An optimized physiologically-based pharmacokinetic (PBPK) model integrated with *CYP3A5* polymorphism was successfully established, providing more insights regarding the long-term DDI between tacrolimus and Wuzhi capsules in patients with different *CYP3A5* genotypes.

## 1. Introduction

The pharmacokinetic drug–drug interaction (DDI) is usually caused by the interference of a perpetrator drug on the metabolizing enzymes or drug transporters of a victim drug. Genetic polymorphisms that alter these enzymes or transporters thereby can affect the magnitude of a DDI. Many clinical cases have demonstrated such influence. For example, tacrolimus metabolism inhibition is significantly greater in renal transplant recipients lacking CYP3A5*1 allele with no functional enzyme activity [1].

**Figure 1:**
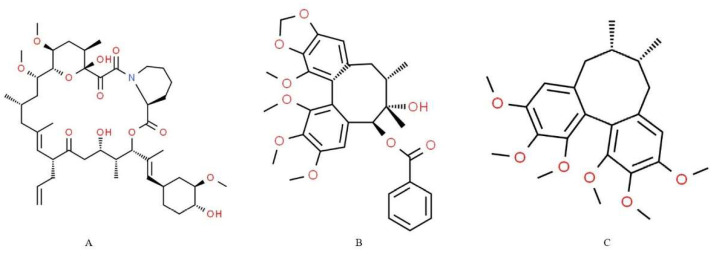
Structures of tacrolimus (**A**), schisantherin A (STA, **B**) and schisandrin A (SIA, **C**). Retrieved from ChemSpider (www.chemspider.com, accessed on 14 Febuaray 2021).

As a first-line immunosuppressive agent for organ transplant patients, tacrolimus is mainly metabolized by CYP3A4 and CYP3A5 [1,2]. Its chemical structure is displayed in Figure 1. An in vitro study has shown that the intrinsic clearance of tacrolimus by CYP3A5 is more than two-fold faster than by CYP3A4 [3]. Moreover, the polymorphism of *CYP3A5* accounts for 40–50% of the variability in tacrolimus dose requirement [4]. The primary determinant of this pharmacogenetic effect is a single-nucleotide polymorphism (SNP) in *CYP3A5* (6986A > G; SNP rs776746), as known as CYP3A5*3 [2]. The presence of the CYP3A5*3 allele is considered “inactive” and classified as a non-expresser phenotype, while the “active” CYP3A5*1 allele infers to *CYP3A5* expresser phenotype [5]. Clotrimazole was found to have a significantly greater inhibition on tacrolimus metabolism in *CYP3A5* non-expresser patients than in *CYP3A5* expressers [2]. However, a contrary trend has been observed for no significant inhibition among *CYP3A5* non-expressers [3], indicating the effect of *CYP3A5* genotype on DDI between tacrolimus and other drugs remains unclear [6].

Wuzhi capsule (WZC), a preparation of Schisandra sphenanthera ethanol extract, is often coadministered with tacrolimus to alleviate drug-induced hepatotoxicity in transplant recipients [7]. WZC contains complex active ingredients. Schisantherin A (STA) and Schisandrin A (SIA), as the two most abundant active ingredients, have shown inhibitory effects on CYP3A activity. Their chemical structures [8] are shown in Figure 1. STA reversibly inhibits CYP3A with an estimated inhibitory constant (K_i_) value of 0.049 μM (the substrate is erythromycin) and inactivates erythromycin N-demethylation in a time–concentration-dependent manner [9]. The maximal inactivation rate constant (k_inact_) and the inhibitor concentration causing half-maximal inactivation (K_I_) values were calculated to be 0.092 min^−1^ and 0.399 μM, respectively [9]. Moreover, in vitro studies [10,11] showed that SIA inhibited CYP3A activity in rat liver microsomes (RLM) with estimated K_i_ values of 5.83 and 4.8 μM for midazolam 1′-hydroxylation and 4-hydroxylation inactivation activity, respectively [11]. Furthermore, in vivo DDI studies in rats indicated that systemic exposure of tacrolimus could be increased 2.48-fold and 2.37-fold due to an oral dose of 0.024 mM/kg STA or SIA, respectively [12]. Clinically, several DDI reports involving 340 patients among eight hospitals showed that the whole-blood trough concentrations of tacrolimus were markedly increased 1.57 to 4.66-fold after WZC administration [13]. Thus, it is crucial to identify the effect of genetic variation on DDI for a better dosing strategy to minimize the occurrence of subtherapeutic or toxic concentrations.

Our previous work developed a valid physiologically-based pharmacokinetic (PBPK) model to quantify the DDI between tacrolimus and WZC without considering *CYP3A5* polymorphism [14]. It was reported that the homozygous wild type (*1/*1) frequency is around 7% in the Asian population, while the heterozygous and homozygous mutant types take up 93% [15]. The Clinical Pharmacogenetics Implementation Consortium (CPIC) guideline for tacrolimus dosing has recommended 1.5 to 2 times higher doses for CYP3A5*1 allele carriers to achieve similar blood concentration levels with CYP3A5 non-expressers [16]. Therefore, in this study we tried to identify how the different genotypes of *CYP3A5* allele could contribute to the variability of interaction between tacrolimus and WZC. The first step was to investigate the inhibitory potency of STA and SIA on tacrolimus metabolism by CYP3A4 and CYP3A5 separately. CYP3cide (PF-04981517), a selective inhibitor of CYP3A4, is commonly used to determine contributions of CYP3A4 and CYP3A5 individually for CYP3A-cleared drugs [17,18,19]. In the present work, we incubated the human liver microsome (HLM) genotyped CYP3A5*1/*3 with CYP3cide to acquire the relevant parameters of CYP3A5, and HLM genotyped CYP3A5*3/*3 without CYP3cide for CYP3A4. The second step was to quantify the contribution of STA and SIA on DDI under different dosing strategies and estimate the impact of *CYP3A5* polymorphism on using tacrolimus and WZC. In this regard, PBPK modeling and simulation was applied to accomplish these objectives by providing a quantitative framework to assess potential DDI [20,21] and integrating the pharmacogenetic factors into the pharmacokinetic change of drugs [22,23,24].

## 2. Results

### 2.1. RI Assay

**Figure 2:**
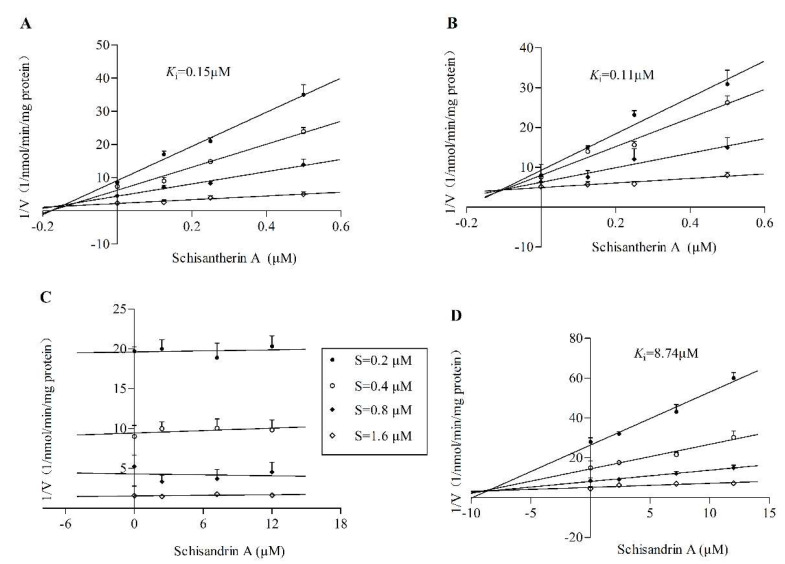
Dixon plots of STA on CYP3A4 (**A**) and CYP3A5 (**B**) of tacrolimus activity metabolism; Dixon plots of SIA on CYP3A4 (**C**) and CYP3A5 (**D**) tacrolimus activity metabolism. Different concentrations of STA (0, 0.125, 0.25 or 0.5 μM), SIA (0, 2.4, 7.2 or 12 μM) and tacrolimus (0.2, 0.4, 0.8 or 1.6 μM) were used. Each incubation was conducted in triplicate (mean values and standard variation (SD) values are listed in Table S1).

Dixon plots yield the K_i_ values for the inhibition by STA on CYP3A4 and CYP3A5 of 0.15 and 0.11 μM, respectively (Figure 2A,B), presenting a competitive reversible inhibition pattern. However, the reaction rate did not change significantly as the SIA’s concentration increased in the incubation with CYP3A5*3/3 HLM (Figure 2C), suggesting little reversible inhibition on CYP3A4 by SIA. The value of K_i_ for the inhibition on CYP3A5 by SIA was 8.74 μM (Figure 2D). Comparing the Ki values of STA and SIA on CYP3A5 shows that STA has a higher inhibitory potency than SIA.

### 2.2. TDI assay

**Figure 3:**
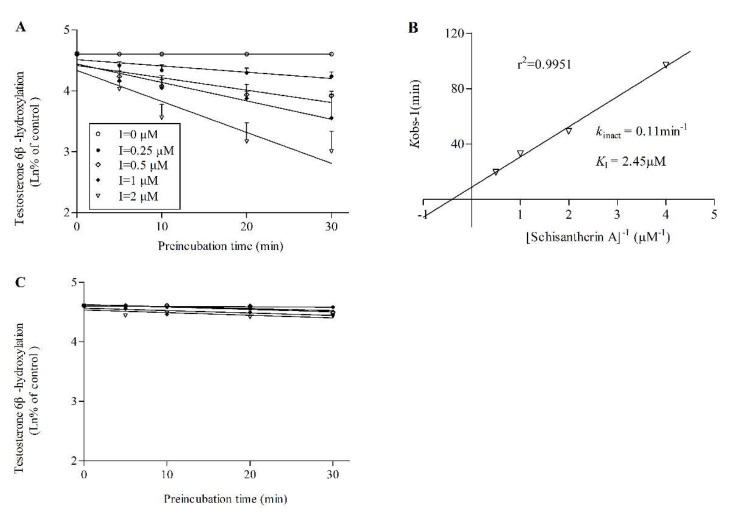
Inactivation of CYP3A4 (**A**,**B**) and CYP3A5(**C**) activity by STA. Various concentrations of STA (0, 0.25, 0.5, 1, 2 μM) and NADPH (Nicotinamide Adenine Dinucleotide Phosphate) at were preincubated at 37 °C for 0, 5, 10, 20 and 30 min in 0.1 M phosphate buffer solution (PBS). (**A**,**C**) a plot of the log of percentage of control activity versus pre-incubation time. (**B**) a plot of the half-life of enzyme inactivation versus the inverse of the STA concentration. Each point represents the average of triplicate experiments (mean and SD values are listed in Table S1).

**Figure 4:**
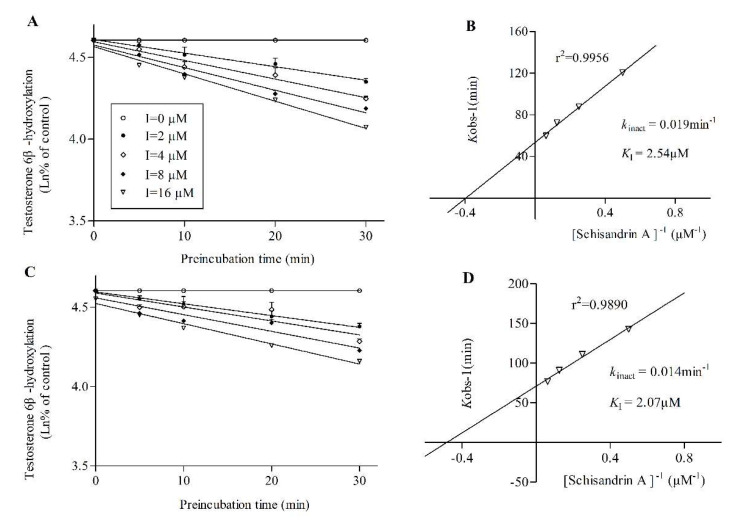
Inactivation of CYP3A4 (**A**,**B**) and CYP3A5 (**C**,**D**) activity by SIA. Various concentrations of SIA (0, 2, 4, 8, 16 μM) and NADPH at were preincubated at 37 °C for 0, 5, 10, 20 and 30 min in 0.1M PBS). (**A**,**C**) a plot of the log of percentage of control activity versus pre-incubation time. (**B**,**D**) a plot of the half-life of enzyme inactivation versus the inverse of the SIA concentration. Each point represents the mean of triplicate experiments (mean and SD values are listed in Table S1).

Testosterone-6β-hydroxylation was used to measure the activity of CYP3A4 and CYP3A5. The logarithm of percent of CYP3A4 or CYP3A5 remaining activity were plotted against pre-incubation time in the presence of various concentrations of inhibitors (STA or SIA). With the corresponding double-reciprocal plot, the derived kinetic constants from the inactivation experiments were estimated. The results indicated that STA showed a potent TDI profile on CYP3A4 (Figure 3A,B) while this was not observed on CYP3A5 (Figure 3C). However, SIA presented a slight TDI on both CYP3A4 (Figure 4A,B) and CYP3A5 (Figure 4C,D) but it was not as strong as STA.

### 2.3. Model Development and Verification

#### 2.3.1. Tacrolimus Pharmacokinetics in CYP3A5 Expressers and Non-Expressers

**Figure 5:**
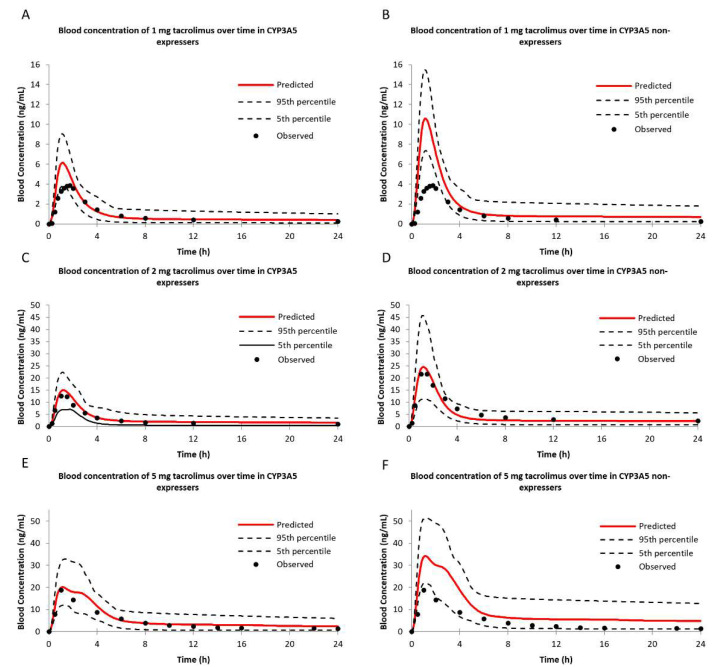
Simulations of blood concentration–time profiles of tacrolimus following a single oral dose of 1 mg (**A**,**B**), 2 mg (**C**,**D**) and 5 mg (**E**,**F**) dose in healthy CYP3A5 expressers and non-expressers by Simcyp^®^. Dashed lines represent the 5th and 95th percentiles of each simulation.

Virtual trials were simulated with the same number of subjects as recruited into the observed study. As shown in Figure 5, simulated blood exposure of tacrolimus after oral administration of different doses (1, 2, 5 mg) in subjects of CYP3A5 expresser and CYP3A5 non-expresser were reasonably consistent with the observed data [1,5,25]. The pharmacokinetics (AUC, *C*_max_, *T*_max_) are listed in Table 1 and all within two-fold values. A good linear correlation (*R*^2^ = 0.9171) between the predicted concentration and the observed concentration was noticed (Figure S1). The predicted AUC of tacrolimus in CYP3A5 non-expressers was much higher than that in CYP3A5 expressers, indicating *CYP3A5* polymorphism could significantly affect the pharmacokinetics of tacrolimus. 

#### 2.3.2. DDI Prediction in CYP3A5 Expressers

Under the circumstance of case #3, the predicted AUC of tacrolimus after a single oral dose of STA was 156.05 ng/mL·h as a 2.17-fold increase, and multidose of STA increased the AUC of tacrolimus 2.70-fold. RI (case #1) and TDI (case #2) by STA increased the AUC of tacrolimus by 1.78- and 1.33-fold, respectively, in the single-dose simulation, and showed 1.86- and 1.76-fold increase in the multidose setting.

#### 2.3.3. DDI Prediction in CYP3A5 Non-Expressers

**Figure 6:**
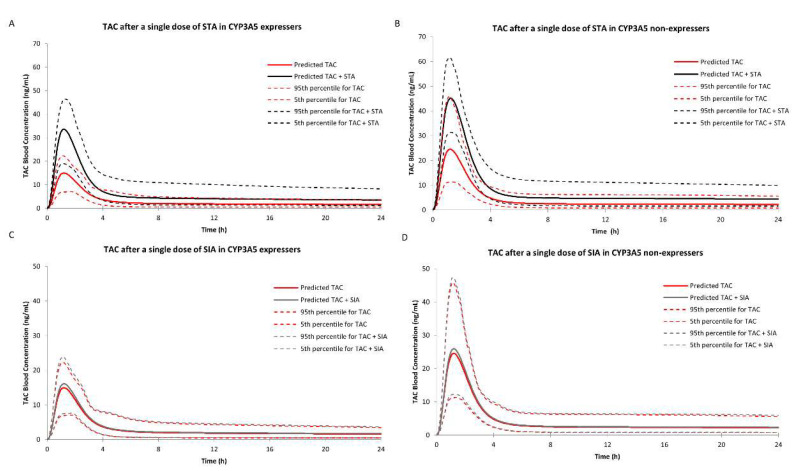
Simulations of blood concentration-time profiles of tacrolimus after a single oral dose of 7.325 mg STA, 7.20 mg SIA in CYP3A5 expressers (**A**,**C**) and CYP3A5 non-expressers (**B**,**D**) by Simcyp^®^.

**Figure 7:**
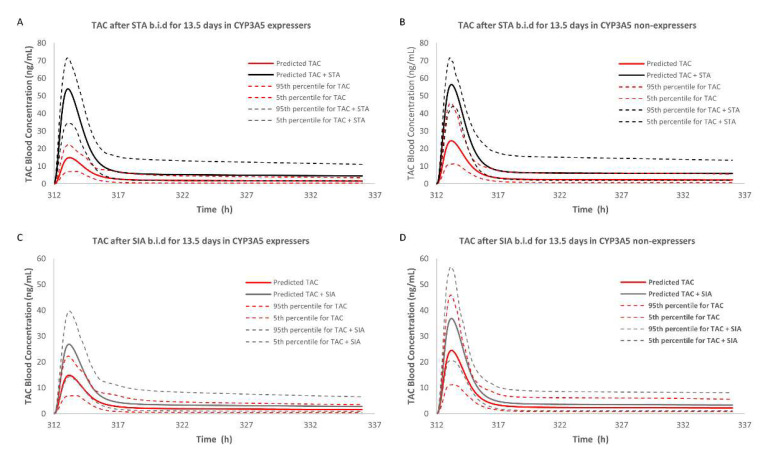
Simulations of blood concentration-time profiles of tacrolimus after multidose of STA (7.325 mg twice daily for 13 days) or SIA (7.2 mg twice daily for 13 days) in CYP3A5 expressers (**A**,**C**) and CYP3A5 non-expressers (**B**,**D**) by Simcyp^®^.

Under the circumstances of case #3, the predicted AUC of tacrolimus in blood after a single oral dose of STA was 195.23 ng/mL·h as a 1.90-fold increase, and the multidose of STA could increase the AUC of tacrolimus 2.41-fold. RI or TDI increased the AUC of tacrolimus 1.48- or 1.50-fold, respectively, in the single-dose simulation, compared with 1.52- and 2.27-fold increases in the multidose setting. On the other hand, for simulation with SIA, there was a small contribution to AUC ratio (AUCR) with the values of 1.10 and 1.39 only via TDI in single-dose and multidose simulation, respectively. All of the above data are presented in Figure 6, Figure 7 and Table 2.

## 3. Discussion

Prior pharmacokinetic studies have noted that WZC increased tacrolimus’s concentration by inhibiting CYP3A4/5 enzymes and our previous work has quantified the contribution of STA and SIA to the interaction from the combined CYP3A4/5 perspective. In the present study, we managed to acquire the inhibitive potency of CYP3A4 and CYP3A5 separately. Although acceptable fold error values (less than two) were noted in all three dosing regimens during tacrolimus model verification, there existed some disparities on the high end in lower dosing (1 mg) and higher dosing (5 mg) groups. Therefore, we decided to use 2 mg dosing in our DDI prediction model to reduce variation. Integrating with the obtained parameters and pharmacogenetic factors, we established an optimized PBPK model to evaluate the DDI between tacrolimus and STA or SIA in patients with two different *CYP3A5* genotypes.

RI and TDI assays were performed with CYP3cide and HLMs of different *CYP3A5* alleles to measure the inhibitory effect of STA or SIA on separate CYP3A enzymes. Our results suggested that STA not only exhibited reversible inhibition on CYP3A4 (*K*_i_ = 0.15 μM) and CYP3A5 (*K*_i_ = 0.11 μM), but also showed robust inactivated inhibition on CYP3A4 (*k*_inact_ = 0.11 min^−1^, *K*_I_ = 2.45 μM, k_inact_/*K*_I_ = 44.90 mL/min/μmol). It was far less than that reported previously (*k*_inact_ = 0.399 min^−1^, *K*_I_ = 0.092 μM, *k*_inact_/*K*_I_ = 230.6 mL/min/μmol) with erythromycin being a probe substrate [9]. Two possible factors could explain the differences. On the one hand, erythromycin itself has been reported to be an irreversible inhibitor of CYP3A using testosterone and midazolam as probe substrates. Moreover, the inhibitory potency of STA from our results was higher than that of verapamil using testosterone as the substrate [26]. On the other hand, this discrepancy could be attributed to the existence of multiple CYP3A binding sites. *In vitro* studies supported the hypothesis of distinct binding domains for each substrate subgroup (midazolam, testosterone, and nifedipine) [27,28]. One important finding was that K_i_ values (0.15 or 0.11 μM) from RI were way less than relevant clinical STA concentration, increasing the likelihood of clinical DDI and toxicity. In comparison with STA, SIA showed a mild RI on CYP3A5 with *K*_i_ values of 8.74 μM while little inhibition on CYP3A4, and a weak TDI on CYP3A4 (k_inact_ = 0.019 min^−1^, *K*_I_ = 2.54 μM, *k*_inact_/*K*_I_ = 7.48 mL/min/μmol) and CYP3A5(*k*_inact_ = 0.014 min^−1^, *K*_I_ = 2.07 μM, *k*_inact_/*K*_I_ = 6.76 mL/min/μmol). The inhibition values were less than the reported ones in rat liver microsome (RLM), which could be explained by the fact of different species of microsome and the types of probe substrate [11]. In brief, this observation confirmed that STA and SIA inhibited CYP3A4 or CYP3A5 under a different mechanism and potency. The acquired parameters could be input into PBPK modeling for more accurate DDI prediction.

After integrating metabolic data and abundance of enzymes into Simcyp^®^ Simulator, plasma concentration-time profiles of tacrolimus under the single dose of 1, 2, and 5 mg in both CYP3A5 expressers and non-expressers matched well with the in vivo profiles. In the DDI study with a single dose of STA, the blood AUCR of tacrolimus was 1.90 in CYP3A5 non-expressers, and the contribution of RI was comparable to TDI (1.48 versus 1.50). However, the AUCR was 2.17 in CYP3A5 expressers and the potency of RI was higher than TDI (1.78 versus 1.33). Similarly, the blood AUCR in CYP3A5 expressers was higher than non-expresser (2.70 versus 2.41) in the multidose setting CYP3A5. It could be concluded that TDI was more prominent in the long-term administration due to its irreversible time-dependent manner. Compared with STA, the blood AUCRs of tacrolimus after SIA use were higher in CYP3A5 expressers than non-expressers in both dosing simulations. A small contribution was observed in the single-dose group, and the multidose use had a moderate one to DDI. Overall, both STA and SIA could increase the blood exposure of tacrolimus. STA showed greater potency than SIA in both inhibition mechanisms, corroborating our previous study. What could be drawn from the present study exclusively was that CYP3A5 showed a lower binding priority than CYP3A4, given the uneven increases among the three cases. It also should be noted that the rises in blood exposure of tacrolimus in CYP3A5 expressers were higher than non-expressers, suggesting the CYP3A5 non-expressers were less prone to inhibitory effects caused by STA or SIA.

Some studies have demonstrated the impact of genetic polymorphism in *CYP3A5* on DDI between tacrolimus and other drugs. It was reported that the trough concentration/dose (C_0_/D) of tacrolimus was significantly increased in renal transplantation adult patients with CYP3A5 expressers after 1, 3, 6, 12 months of WZC use, while it had no statistical significance in CYP3A5 non-expressers [29]. Similarly, the oral clearance of tacrolimus was decreased 2.2-fold when coadministered with amlodipine in CYP3A5 expressers but not in non-expressers [30]. Our result was consistent with these studies. Given the mutation of CYP3A5*3 resulting in the expression of inactive proteins, *CYP3A5* genotype-dependent inhibition identified in the present study could be explained. However, the other studies have come to the opposite conclusion. Renal transplant pediatric patients with CYP3A5 non-expressers suffering from nephrotic syndrome showed a higher blood concentration of tacrolimus than CYP3A5 expressers after one-week WZC use [31]. Moreover, ketoconazole exhibited a more significant inhibition with a higher tacrolimus level in CYP3A5 non-expressers than expressers [32]. A possible explanation for these findings could be specific to inhibitors, but the exact mechanism behind this controversy remains to be further explored.

As tacrolimus is expensive and requires long-term use while WZC is affordable, the coadministration could cut down the cost of treatment by 40–60% for each patient per year [33]. Applying the DDI prediction framework concluded from the current work could possibly serve as a dosing guidance tool. However, several limitations to this study need to be acknowledged beforehand. First, only healthy male patients were generated in our simulation in order to match the clinical data. Not including transplant patients might bias the pharmacokinetic characteristics since different pathophysiological conditions could alter the drugs’ disposition. Further investigation should consider specific transplant patient population pharmacokinetics characteristics. Second, our final DDI prediction was only based on a single individual component, lacking clinical feasibility because either STA or SIA was not used alone clinically but WZC as a whole. Due to the complex nature of WZC ingredients and lacking guidelines on manufacturing, the inappropriate clinical use of WZC is prone to inconsistent efficacy and toxicity, posting a challenge on investigations by clinical trials. Separating and quantifying each component’s contribution in vivo is difficult but still needed for product standardization. Through the PBPK model we built, an individual component’s pharmacokinetic properties could be simulated, validated, and further extrapolated to in vivo to help tackle these barriers. Further optimization on our PBPK model to integrate more active components into the algorithm could improve clinical relevance. Third, our prediction results require further validation from real-world data to implement the findings despite the increased systemic exposure noticed in the result. Although we are not confident enough to suggest avoiding WZC on tacrolimus patients at this moment, therapeutic drug monitoring (TDM) is strongly recommended.

One important future direction of individualized medicine is how to interpret and utilize pharmacogenomic variation. Knowledge of *CYP3A5* polymorphism could help understand the pharmacokinetics of tacrolimus with WZC use. Patients with CYP3A5 expressers exhibit a much greater inhibition by the two principal ingredients (STA and SIA) of WZC. Thus, our study supports a strategy to evaluate the inhibitive potency on different *CYP3A5* genotypes and integrate the information into PBPK modeling to investigate the magnitude of DDI.

## 4. Materials and Methods

### 4.1. Chemicals and Reagents

Schisantherin A (purity ≥ 99.0%, lot: PS12102902) and schisandrin A (purity ≥ 99.0%, lot: PS12102301) were obtained from PUSH Bio-Technology Co., Ltd. (Chengdu, China). Tacrolimus (purity ≥ 99.0%, lot: I1507118) was from Aladdin Biochemical Technology Co., Ltd. (Shanghai, China). Ascomycin (purity ≥ 99.0%, lot: D1202AS) and prednisolone (purity ≥ 98.0%, lot: J0402AS) were purchased from Dalian Meilun Bio-Technology Co., Ltd. (Dalian, China); CYP3cide (PF-4981517, lot: ECD 192-1-PFZ) was obtained from J&K Technology Co., Ltd. (Beijing, China). CYP3A5*1/*3 HLM (lot: 0710232) and CYP3A5*3/*3 HLM (lot: 0710253) were obtained from Transheep Co., Ltd. (Shanghai, China). All other chemicals and reagents were the same as described in our previous study [14].

### 4.2. HPLC-MS/MS Method

Tacrolimus and 6β-hydroxyl-testosterone in all samples were detected using HPLC-MS/MS methods in our published study [14]. In brief, Multiple Reaction Monitoring (MRM) mode and positive ion mode were performed. Mass transitions (*m*/*z*), declustering potential (DP) and collision energy (CE) for detection were shown in Table S2. For tacrolimus and its internal standard ascomycin, the mobile phase was methanol–10mM ammonium acetate with 0.1% acetic acid (95:5). For 6β-hydroxyl-testosterone and its internal standard prednisolone, the mobile phase was acetonitrile–0.1% formic acid (60:40). The flow rate of the mobile phase was set at 0.3 mL/min. The linearity of quantifications was in the range of 0.05–4 μM and 0.1953–12.5 μM for tacrolimus (*R*^2^ = 0.9995) and 6β-hydroxyl-testosterone (*R*^2^ = 1.000), respectively.

### 4.3. Reversible Inhibition (RI) Assay of STA/SIA on CYP3A4 and CYP3A5

In this study, CYP3A5*3/*3 HLM was used to study the inhibition mechanism of STA/SIA on CYP3A4. Similarly, CYP3A5*1/*3 HLM along with CYP3cide was chosen to determine the inhibition mechanism of STA/SIA on CYP3A5. In order to obtain the K_i_ value of STA/SIA on CYP3A4, different concentrations of tacrolimus (0.2, 0.4, 0.8, 1.6 μM) together with STA (0, 0.125, 0.25, 0.5 μM) or SIA (0, 2.4, 7.2, 12 μM) were pre-incubated with CYP3A5*3/*3 HLM (0.2 mg/mL) and 0.1 M potassium phosphate buffer solution (PBS, pH = 7.4) for 3 min in a shaking water bath at 37℃. The reactions were started with the addition of nicotinamide adenine dinucleotide phosphate (NADPH, 1 mM) and stopped 10 min later upon adding a double volume of ice-cold methanol. For the K_i_ value of CYP3A5, CYP3cide with a final concentration of 0.5 μM and CYP3A5*1/*3 HLM with a final concentration of 0.2 mg/mL were pre-incubated for 10 min in the presence of NADPH (1 mM) before tacrolimus and STA or SIA were added to the mixture. Reactions were terminated with the same method as the CYP3A4 group. All incubations were carried out in triplicate. Samples were processed and detected as described in our previously published article [12]. Dixon plots were used to analyze the inhibitory type of RI assay and K_i_ value.

### 4.4. Time-Dependent Inhibition (TDI) Assay of STA/SIA on CYP3A4 and CYP3A5

Two-step incubation was performed to evaluate the TDI assay. First step, for TDI on CYP3A4, CYP3A5*3/*3 HLM (0.5 mg/mL) was added to the incubation with diverse concentrations of STA (0, 0.25, 0.5, 1, 2 μM) or SIA (0, 2, 4, 8, 16 μM). After pre-incubation for 3 min at 37 °C in a shaking water bath, the enzymatic reaction was started upon adding NADPH (1 mM) with various pre-reaction time (0, 5, 10, 20, 30 min). For TDI on CYP3A5, NADPH (1 mM) was added to the incubation which contained CYP3A5*1/*3 HLM (0.5 mg/mL) and CYP3cide (1.2 μM) after being pre-warmed for 3 min. The mixture was added with different concentrations of STA or SIA 10 min later and shared the same various incubation times as the CYP3A4 group. Next step, an aliquot (20 μL) of the primary pre-reaction mixture was transferred to 180 μL of the secondary incubation system which consisted of PBS (0.1 M), testosterone (200 μM) and NADPH (1 mM). The secondary reaction was incubated at 37 °C for 10 min and terminated by adding 400 μL ice-cold acetonitrile containing prednisolone as the internal standard (0.3150 μM for CYP3A5*3/*3 HLM incubation and 0.07875 μM for CYP3A5*1/*3 HLM incubation). In order to calculate the inactivation kinetic parameters, a linear regression of the logarithm of “the percentage of formation rate of 6β-hydroxyl-testosterone” against the pre-incubation time in the function of various concentrations of inhibitors (STA or SIA) was applied. The observed inactivation rate of the affected enzyme (*k*_obs_) was the negative slope of the linear regression. *k*_inact_ and *K*_I_ were calculated using Equation (1) [34] by the double-reciprocal plot. *I* represents the concentration of the inhibitor.

(1) $$k_{\mathrm{obs}}=\frac{k_{\mathrm{inact}}\times I}{K_{I}\times I}$$

All incubations were carried out in triplicate. Samples were processed and detected as described in our previously published article [14].

### 4.5. PBPK Model Development of Tacrolimus, STA and SIA

All PBPK simulations were constructed using the population-based ADME(absorption, distribution, metabolism, and excretion) simulator (version 13.1.1, Simcyp^®^ Ltd., Certara, Sheffield, UK). The drug-related properties including physicochemical and in vitro PK parameters were listed in Tables S3 and S4 and used to build PBPK models of tacrolimus, STA, and SIA.

Tacrolimus human PBPK models of CYP3A5 expressers and non-expressers were slightly modified based on the model we published before [14]. The intrinsic clearance values of each CYP isoform were listed in Table 3. Two virtual genotype populations, one for CYP3A5 expressers and the other for non-expressers, were manually created from the built-in healthy Chinese and Caucasian populations. The default abundance settings of CYP3A4 and CYP3A5 in Chinese and Caucasian are listed in Table 4. As part of the model optimization, we adapted the sirolimus PBPK modeling method reported in the literature [35]. The tissue-to-plasma partition coefficient (*K*_p_) value of each tissue was tested at 10, 100, and 1000 as a default setting for screening purposes to identify the most significant contribution to tacrolimus tissue distribution volume. The result showed the *K*_p_ of adipose tissue set at the maximum value improved model performance, while there was no evident effect for any other tissues such as brain, gut, heart, kidney, lung, or liver. The sensitivity analysis of the *K*_p_ value of adipose tissue ranging from 250 to 1000 indicated the best fitting at 1000, which specifically reflected the distribution phase of CYP3A5 expressers and non-expressers (Figure S2, Table S5). The establishment of PBPK models of STA and SIA was the same as in our prior study [14].

### 4.6. Simcyp^®^ Simulations

All simulation trials were conducted with a healthy virtual population (fasted state) derived from the clinical study [1,5,25]. The plasma concentration profiles of tacrolimus in CYP3A5 expressers and non-expressers were simulated with a single oral dose of 1, 2, and 5 mg, respectively. The dose regimens, basic biological information, and numbers of patients were the same as in our prior study [14]. The demographic characteristics of patients are listed in Table S6. Predicted values of pharmacokinetic parameters including area under the plasma concentration-time curve (AUC), total maximal concentration in plasma (C_max_), and time to reach the maximal concentration in plasma (T_max_) were assessed against the observed data using Equation (2).

(2) $$\mathrm{Fold}\;\mathrm{error}=\frac{\mathrm{predicted}\;\mathrm{value}}{\mathrm{obersved}\;\mathrm{value}}\;(\mathrm{if}\;\mathrm{predicted}\;>\;\mathrm{observed})\;\mathrm{Or}\;\mathrm{Fold}\;\mathrm{error}=\frac{\mathrm{observed}\;\mathrm{value}}{\mathrm{predicted}\;\mathrm{value}}\;(\mathrm{if}\;\mathrm{observed}\;>\;\mathrm{predicted})$$

The model is considered to have good fitness if the fold error is less than 2 [36]. The DDI between tacrolimus and STA or SIA in CYP3A5 expressers and CYP3A5 non-expressers of healthy Chinese populations was simulated using the established PBPK model with 2 mg tacrolimus. *K*_i_, *K*_I_, and *k*_inact_ values of CYP3A4 and CYP3A5 by STA and SIA were integrated into this DDI model. To determine the contributions of RI and TDI to fold-increase in tacrolimus AUC in CYP3A5 expressers and non-expressers, we simulated three cases with different inhibitions: case #1 with only RI, case #2 with only TDI, case #3 with combined RI and TDI.

## 5. Conclusions

Based on our previous work, the present study aimed to distinguish STA or SIA’s inhibitory effect on CYP3A4 and CYP3A5 of different genotypes. An optimized PBPK model integrated with *morphism was successfully established, providing more insights to evaluate DDI between tacrolimus and Wuzhi capsule in patients with different *CYP3A5* genotypes regarding the long-term combination use.

## Figures and Tables

**Table 1:** Simulation results of tacrolimus with different doses.

Parameters		*C*_max_ (ng/mL)			*T*_max_ (h)			AUC (ng/mL·h)		
Dose	Population	Pre ^3^	Obs ^4^	FE ^5^	Pre	Obs	FE	Pre	Obs	FE
1 mg	Exp ^1^ (*n* = 16)	5.74	3.88	1.48	1.08	1.75	1.61	22.50	19.34	1.16
	Non-exp ^2^ (*n* = 26)	9.93	5.52	1.80	1.08	1.5	1.38	37.47	30.34	1.24
2 mg	Exp (*n* = 31)	14.50	14.09	1.03	1.08	1.26	1.16	71.53	60.83	1.18
	Non-exp (*n* = 40)	23.87	24.28	1.02	1.08	1.35	1.25	102.54	119.02	1.16
5 mg	Exp (*n* = 12)	21.41	20.8	1.03	0.84	1.40	1.67	131.07	90.40	1.45
	Non-exp (*n* = 12)	36.73	27.90	1.32	0.84	1.30	1.55	227.07	134.77	1.68

^1^ Exp: CYP3A5 expressers; ^2^ Non-exp: CYP3A5 non-expressers; ^3^ Pre: Predicted; ^4^ Obs: Observed; ^5^ FE: Fold error.

**Table 2:** Results of drug-drug interaction (DDI) prediction for single-dose and multidose of STA and SIA in CYP3A5 expressers and non-expressers.

	AUCR	Inhibitors	Dose Regimen	RI Case#1	TDI Case#2	RI and TDI Case#3
Population						
CYP3A5 Non-expresser		STA	Single dose	102.80 ^1^/152.07 ^2^ 1.48 ^3^	102.80/154.70 1.50	102.80/195.23 1.90
			Multidose	102.81/156.70 1.52	102.81/233.02 2.27	102.81/247.91 2.41
		SIA	Single dose	—	102.80/113.25 1.10	—
			Multidose	—	102.81/142.93 1.39	—
CYP3A5 Expresser		STA	Single dose	72.04/127.91 1.78	72.04/65.67 1.33	72.04/156.05 2.17
			Multidose	72.05/134.10 1.86	72.05/126.94 1.76	72.05/193.91 2.70
		SIA	Single dose	72.04/73.20 1.02	72.04/80.99 1.12	72.04/82.24 1.14
			Multidose	72.05/73.20 1.02	72.05/111.45 1.55	72.05/112.85 1.57

^1^ the predicted area under the plasma concentration-time curve (AUC) of tacrolimus alone; ^2^ the predicted AUC of tacrolimus after coadministered with inhibitors (unit, ng/mL·h); ^3^ AUCR (AUC ratio), stands for fold-increase in AUC by an interacting drug.

**Table 3:** Intrinsic clearance values for tacrolimus in each CYP isoform.

Elimination	Parameters	Value	Source
CYP3A5 Expresser	CYP3A4/5 13-DMT ^1^ V_max_^2^	8/17 pmol/min/pmol	[3]
	CYP3A4/5 13-DMT K_m,u_^3^	0.21/0.21 μM	
	CYP3A4/5 12-HT ^4^ V_max_	0.6/1.4 pmol/min/pmol	[3]
	CYP3A4/5 12-HT K_m,u_	0.29/0.35 μM	[3]
CYP3A5 Non-expresser	CYP3A4 13-DMT/12 HT V_max_	8/0.6 pmol/min/pmol	[3]
	CYP3A4 13-DMT/12-HT K_m,u_	0.21/0.29 μM	[3]
	CYP3A4/5 ISEF ^5^	0.24 (BD Supersomes)	Simcyp^®^

^1^ 13-DMT: 13-O-desmethyl tacrolimus; ^2^ Vmax: maximal rate; ^3^ K_m,u_: unbound Michaelis constant; ^4^ 12-HT: 12-hydroxy tacrolimus; ^5^ ISEF: inter-system extrapolation factors.

**Table 4:** CYP3A4 and CYP3A5 abundance in two virtual genotype populations.

Population	CYP3A4/5	Caucasian	Chinese
CYP3A5 expressers	CYP3A4 in liver	137	120
	CYP3A5 in liver	103	82
	CYP3A4 in intestine	66.2	58
	CYP3A5 in intestine	24.6	21.5
CYP3A5 non-expressers	CYP3A4 in liver	137	120
	CYP3A4 in intestine	66.2	58
	CYP3A5 in liver and intestine	0	0

## Data Availability

Data is contained within the article or supplementary material.

## Supplementary Materials

The following are available online at https://www.mdpi.com/1424-8247/14/3/198/s1, Figure S1: Linear analysis of the predicted concentration and observed concentration of tacrolimus; Figure S2: Sensitivity analysis of adipose distribution on tacrolimus pharmacokinetics in CYP3A5 expressers and non-expressers; Table S1: Data points for RI and TDI assays with mean and standard variation values; Table S2: Optimized detection parameters and LC-MS/MS conditions; Table S3: Parameters used for PBPK modeling of schisantherin A and schisandrin A; Table S4: Parameters used for PBPK modeling of tacrolimus; Table S5: C_max_ and AUC of tacrolimus under different *K*_p_ values; Table S6: Demographic information used for PBPK modeling of tacrolimus.

## References

[1] Choi Y., Jiang F., An H., Park H.J., Choi J.H., Lee H. (2017). A pharmacogenomic study on the pharmacokinetics of tacrolimus in healthy subjects using the DMETTM Plus platform. Pharm. J..

[2] Coto E., Tavira B., Suárez-Álvarez B., López-Larrea C., Díaz-Corte C., Ortega F., Álvarez V. (2011). Pharmacogenetics of tacrolimus: Ready for clinical translation?. Kidney Int..

[3] Dai Y., Hebert M.F., Isoherranen N., Davis C.L., Marsh C., Shen D.D., Thummel K.E. (2006). Effect of CYP3A5 polymorphism on tacrolimus metabolic clearance in vitro. Drug Metab. Dispos..

[4] Tang J.T., Andrews L.M., van Gelder T., Shi Y.Y., van Schaik R.H.N., Wang L.L., Hesselink D.A. (2016). Pharmacogenetic aspects of the use of tacrolimus in renal transplantation: Recent developments and ethnic considerations. Expert Opin. Drug Metab. Toxicol..

[5] Zheng S., Tasnif Y., Hebert M.F., Davis C.L., Shitara Y., Calamia J.C., Lin Y.S., Shen D.D., Thummel K.E. (2012). Measurement and compartmental modeling of the effect of CYP3A5 gene variation on systemic and intrarenal tacrolimus disposition. Clin. Pharmacol. Ther..

[6] Obach R.S., Walsky R.L., Venkatakrishnan K. (2007). Mechanism-based inactivation of human cytochrome P450 enzymes and the prediction of drug-drug interactions. Drug Metab. Dispos..

[7] Xin H.-W., Wu X.-C., Li Q., Yu A.-R., Zhu M., Shen Y., Su D., Xiong L. (2007). Effects of Schisandra sphenanthera extract on the pharmacokinetics of tacrolimus in healthy volunteers. Br. J. Clin. Pharmacol..

[8] ChemSpider. http://www.chemspider.com/.

[9] Iwata H., Tezuka Y., Kadota S., Hiratsuka A., Watabe T. (2004). Identification and characterization of potent CYP3A4 inhibitors in schisandra fruit extract. Drug Metab. Dispos..

[10] Lai L., Hao H., Wang Q., Zheng C., Zhou F., Liu Y., Wang Y., Yu G., Kang A., Peng Y. (2009). Effects of short-term and long-term pretreatment of schisandra lignans on regulating hepatic and intestinal CYP3A in Rats. Drug Metab. Dispos..

[11] Li W.-L., Xin H.-W., Su M.-W. (2012). Inhibitory Effects of Continuous Ingestion of Schisandrin A on CYP3A in the Rat. Basic Clin. Pharmacol. Toxicol..

[12] Qin X.L., Chen X., Wang Y., Xue X.P., Wang Y., Li J.L., Wang X.D., Zhong G.P., Wang C.X., Yang H. (2014). In vivo to in vitro effects of six bioactive lignans of Wuzhi tablet (schisandra sphenanthera extract) on the CYP3A/P-glycoprotein–mediated absorption and metabolism of tacrolimus. Drug Metab. Dispos..

[13] Chen T., Lu J. (2012). Hyperkalemia induced by tacrolimus combined with Wuzhi-capsule following renal transplantation: One case report (in Chinese). J. Clin. Rehabil. Tissue Eng. Res..

[14] Zhang H., Bu F., Li L., Jiao Z., Ma G., Cai W., Zhuang X., Lin H.-S., Shin J.-G., Xiang X. (2018). Prediction of drug-drug interaction between tacrolimus and principal ingredients of Wuzhi capsule in chinese healthy volunteers using physiologically-based pharmacokinetic modelling. Basic Clin. Pharmacol. Toxicol..

[15] Fukuen S., Maune H., Ikenaga Y., Yamamoto I., Inaba T., Azuma J. (2002). Novel detection assay by PCR–RFLP and frequency of the CYP3A5 SNPs, CYP3A5*3 and *6, in a Japanese population. Pharmacogenetics.

[16] Birdwell K., Decker B., Barbarino J., Peterson J., Stein C., Sadee W., Wang D., Vinks A., He Y., Swen J. (2015). Clinical pharmacogenetics implementation consortium (CPIC) guidelines for CYP3A5 genotype and tacrolimus dosing. Clin. Pharmacol. Toxicol..

[17] Tseng E., Walsky R.L., Luzietti R.A., Harris J.J., Kosa R.E., Goosen T.C., Zientek M.A., Obach R.S. (2014). Relative contributions of cytochrome CYP3A4 versus CYP3A5 for CYP3A-cleared drugs assessed in vitro using a CYP3A4-selective inactivator (CYP3cide). Drug Metab. Dispos..

[18] Walsky R.L., Obach R.S., Hyland R., Kang P., Zhou S., West M., Geoghegan K.F., Helal C.J., Walker G.S., Goosen T.C. (2012). Selective mechanism-based inactivation of CYP3A4 by CYP3cide (PF-04981517) and its utility as an in vitro tool for delineating the relative roles of CYP3A4 versus CYP3A5 in the metabolism of drugs. Drug Metab. Dispos..

[19] Zientek M.A., Goosen T.C., Tseng E., Lin J., Bauman J.N., Walker G.S., Kang P., Jiang Y., Freiwald S., Neul D. (2015). In vitro kinetic characterization of axitinib metabolism. Drug Metab. Dispos..

[20] Yamazaki S., Johnson T.R., Smith B.J. (2015). Prediction of drug-drug interactions with crizotinib as the CYP3A substrate using a physiologically based pharmacokinetic model. Drug Metab. Dispos..

[21] Yu Y., Loi C.M., Hoffman J., Wang D. (2017). Physiologically based pharmacokinetic modeling of palbociclib. J. Clin. Pharmacol..

[22] Djebli N., Fabre D., Boulenc X., Fabre G., Sultan E., Hurbin F. (2015). Physiologically based pharmacokinetic modeling for sequential metabolism: Effect of cyp2c19 genetic polymorphism on clopidogrel and clopidogrel active metabolite pharmacokinetics. Drug Metab. Dispos..

[23] Emoto C., Fukuda T., Venkatasubramanian R., Vinks A.A. (2015). The impact of CYP3A5*3 polymorphism on sirolimus pharmacokinetics: Insights from predictions with a physiologically-based pharmacokinetic model. Br. J. Clin. Pharmacol..

[24] Yeo K.R., Kenny J.R., Rostami-Hodjegan A. (2013). Application of in vitro-in vivo extrapolation (IVIVE) and physiologically based pharmacokinetic (PBPK) modelling to investigate the impact of the CYP2C8 polymorphism on rosiglitazone exposure. Eur. J. Clin. Pharmacol..

[25] Zhang J.-J., Zhang H., Ding X.-L., Ma S., Miao L.-Y. (2013). Effect of the P450 oxidoreductase *28 polymorphism on the pharmacokinetics of tacrolimus in Chinese healthy male volunteers. Eur. J. Clin. Pharmacol..

[26] Shen L., Fitzloff J.F., Cook C.S. (2004). Differential enantioselectivity and product-dependent activation and inhibition in metabolism of verapamil by human CYP3AS. Drug Metab. Dispos..

[27] Yang Y., Liu F., Xiong L., Li W., Yu A. (2017). Study on the association of synergistic effects of Wuzhi capsules on tacrolimus with CYP3A5*3 gene polymorphism (in Chinese). China Pharm..

[28] Zuo X.-C., Zhou Y.-N., Zhang B.-K., Yang G.-P., Cheng Z.-N., Yuan H., Ouyang D.-S., Liu S.-K., Barrett J.S., Li P.-J. (2013). Effect of CYP3A5*3 Polymorphism on Pharmacokinetic Drug Interaction between Tacrolimus and Amlodipine. Drug Metab. Dispos..

[29] Tao S., Peng H., Xia Z. (2016). Influence of Wuzhi-capsule on blood drug concentration of tacrolimus in nephrotic syndrome children with different CYP3A5 genotypes (in Chinese). Jiangsu Med..

[30] Chandel N., Aggarwal P.K., Minz M., Sakhuja V., Kohli K.K., Jha V. (2009). CYP3A5*1/*3 genotype influences the blood concentration of tacrolimus in response to metabolic inhibition by ketoconazole. Pharm. Genom..

[31] Barter Z.E., Perrett H.F., Yeo K.R., Allorge D., Lennard M.S., Rostami-Hodjegan A. (2010). Determination of a quantitative relationship between hepatic CYP3A5*1/*3 and CYP3A4 expression for use in the prediction of metabolic clearance in virtual populations. Biopharm. Drug Dispos..

[32] Tamura S., Tokunaga Y., Ibuki R., Amidon G.L., Sezaki H., Yamashita S. (2003). The site-specific transport and metabolism of tacrolimus in rat small intestine. J. Pharmacol. Exp. Ther..

[33] Xin H., Wu X., He Y., Yu A., Xiong L., Xiong Y. (2011). Evaluation the effects and cost on the application of tacrolimus combination with Wuzhi-capsule in renal transplanted recipients. Chin. J. Clin. Pharmacol..

[34] Hollenberg P.F., Kent U.M., Bumpus N.N. (2008). Mechanism-based inactivation of human cytochromes P450s: Experimental characterization, reactive intermediates, and clinical implications. Chem. Res. Toxicol..

[35] Emoto C., Fukuda T., Cox S., Christians U., Vinks A. (2013). Development of a physiologically-based pharmacokinetic model for sirolimus: Predicting bioavailability based on intestinal CYP3A content. CPT Pharmacomet. Syst. Pharmacol..

[36] Lancia P., Jacqz-Aigrain E., Zhao W. (2015). Choosing the right dose of tacrolimus. Arch. Dis. Child..

