# Supplementary material for: Investigation of the Impact of CYP3A5 Polymorphism on Drug–Drug Interaction between Tacrolimus and Schisantherin A/Schisandrin A Based on Physiologically-Based Pharmacokinetic Modeling
Source: Pharmaceuticals (Basel). 2021 Feb 27;14(3):198. doi: 10.3390/ph14030198 (PMC7997453; doi:10.3390/ph14030198)
Supplement: Supplementary file 1 [file pharmaceuticals-14-00198-s001.pdf]

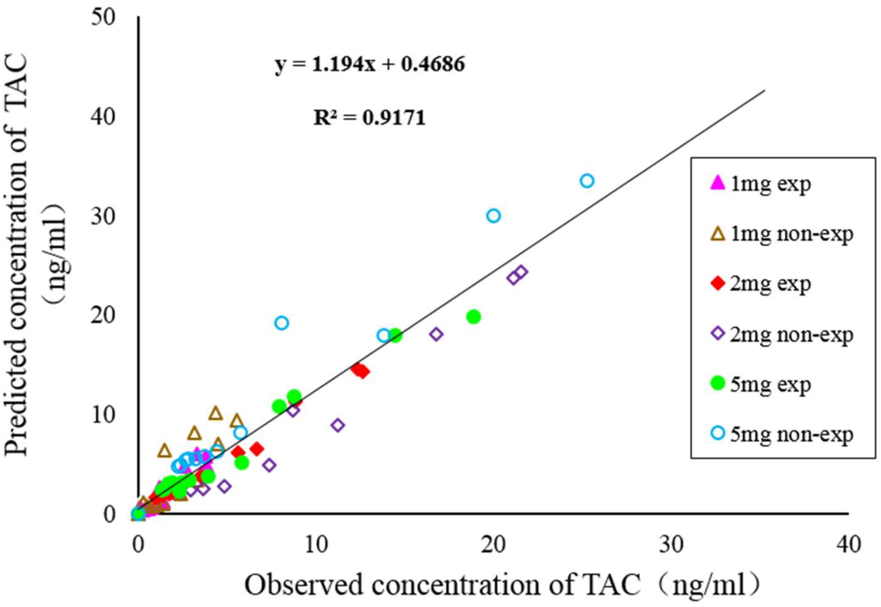

Figure S1. Linear analysis of the predicted concentration and observed concentration of tacrolimus

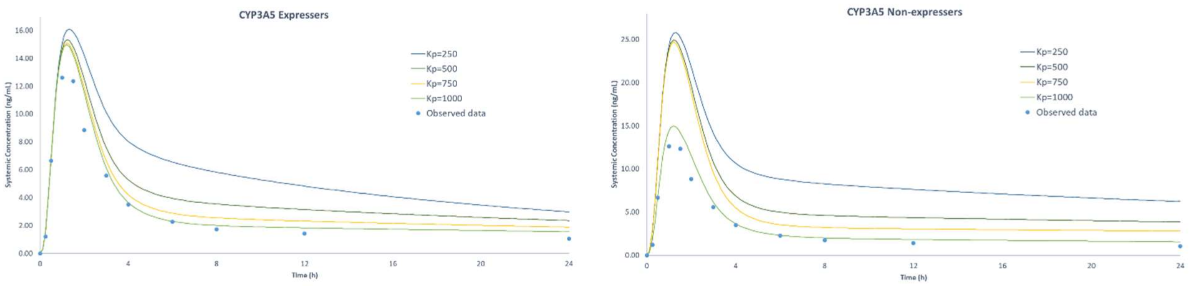

Figure S2. Sensitivity analysis of adipose distribution on tacrolimus pharmacokinetics in CYP3A5 expressers and non-expressers. Blood concentration of tacrolimus following a single oral dose of 2 mg was simulated with K_p value of adipose tissue varying from 250 to 1000 by 250.

Table S1 Data points for RI and TDI assays with mean and standard variation values

Figure 2A								
	**0.2 µM tacrolimus**		**0.4 µM tacrolimus**		**0.8 µM tacrolimus**		**1.6 µM tacrolimus**	
**STA**	**Mean**	**SD**	**Mean**	**SD**	**Mean**	**SD**	**Mean**	**SD**
0 µM	8.5682	0.1101	7.3548	1.2723	4.5004	0.1763	2.3328	0.0625
0.125 µM	17.0000	1.0000	9.0441	1.0494	7.1353	0.6206	2.6233	0.6790
0.25 µM	21.0000	1.0000	14.8861	0.4893	8.3071	0.6418	3.9072	0.6564
0.5 µM	35.0000	3.0000	23.9051	1.2189	13.9244	1.6474	5.0160	0.7723
**Figure 2B**								
	**0.2 µM tacrolimus**		**0.4 µM tacrolimus**		**0.8 µM tacrolimus**		**1.6 µM tacrolimus**	
**STA**	**Mean**	**SD**	**Mean**	**SD**	**Mean**	**SD**	**Mean**	**SD**
0 µM	8.0000	2.7759	7.5682	0.1101	6.3548	1.2723	5.1363	1.1036
0.125 µM	15.0000	0.3663	14.0109	1.4711	7.5773	1.6034	5.6992	0.6790
0.25 µM	23.2012	1.0138	15.6487	0.8529	12.0521	2.6740	5.8700	0.6564
0.5 µM	30.8928	3.5033	26.2841	1.6422	14.9975	2.4334	8.0120	0.7723
**Figure 2C**								
	**0.2 µM tacrolimus**		**0.4 µM tacrolimus**		**0.8 µM tacrolimus**		**1.6 µM tacrolimus**	
**SIA**	**Mean**	**SD**	**Mean**	**SD**	**Mean**	**SD**	**Mean**	**SD**
0 µM	19.7089	0.5288	9.0000	1.4052	5.2438	1.4052	1.5764	1.1963
2.4 µM	20.0000	1.1414	10.0000	0.8362	3.3452	0.8362	1.4662	0.0139
7.2 µM	18.8974	1.8284	10.0565	1.1621	3.6950	1.1621	1.7598	0.1414
12 µM	20.3445	1.2828	9.8175	1.2527	4.5304	1.2327	1.6431	0.0012
**Figure 2D**								
	**0.2 µM tacrolimus**		**0.4 µM tacrolimus**		**0.8 µM tacrolimus**		**1.6 µM tacrolimus**	
**SIA**	**Mean**	**SD**	**Mean**	**SD**	**Mean**	**SD**	**Mean**	**SD**
0 µM	28.0000	2.0288	14.9302	3.4052	8.4000	1.4052	4.4803	1.1963
2.4 µM	32.0000	1.1414	17.6167	0.8362	9.2000	0.8362	6.2790	0.0139
7.2 µM	43.0000	3.8284	21.6176	1.1621	12.0300	1.1621	6.9243	0.1414
12 µM	60.0000	2.8284	30.1990	3.2527	15.0500	1.2327	7.2381	0.0012

Figure 3A										
	2 µM STA		1 µM STA		0.5 µM STA		0.25 µM STA		0 µM STA	
**Time**	**Mean**	**SD**	**Mean**	**SD**	**Mean**	**SD**	**Mean**	**SD**	**Mean**	**SD**
30 min	3.0051	0.3321	3.5554	0.3885	3.9183	0.0745	4.2407	0.0685	4.6052	0.0000
20 min	3.1694	0.3080	3.8785	0.2297	3.9359	0.0519	4.2986	0.0755	4.6052	0.0000
10 min	3.5615	0.2198	4.0515	0.1355	4.0778	0.1705	4.3386	0.0879	4.6052	0.0000
5 min	4.0322	0.1220	4.1658	0.1597	4.2393	0.1871	4.4164	0.0627	4.6052	0.0000
0 min	4.6052	0.0000	4.5902	0.0263	4.6010	0.0073	4.6010	0.0087	4.6052	0.0000
**Figure 4A**										
	16 µM SIA		8 µM SIA		4 µM SIA		2 µM SIA		0 µM SIA	
**Time**	**Mean**	**SD**	**Mean**	**SD**	**Mean**	**SD**	**Mean**	**SD**	**Mean**	**SD**
30 min	4.0738	0.0015	4.1876	0.0077	4.2490	0.0142	4.3520	0.0200	4.6052	0.0000

20 min	4.2397	0.0074	4.2781	0.0201	4.3925	0.0448	4.4602	0.0351	4.6052	0.0000
10 min	4.3776	0.0019	4.3979	0.0093	4.4420	0.0329	4.5165	0.0465	4.6052	0.0000
5 min	4.4518	0.0063	4.5164	0.0047	4.5505	0.0164	4.5754	0.0186	4.6052	0.0000
0 min	4.6052	0.0000	4.6029	0.0023	4.6052	0.0000	4.6052	0.0000	4.6052	0.0000
**Figure 4C**										
	16 μM SIA		8 μM SIA		4 μM SIA		2 μM SIA		0 μM SIA	
**Time**	**Mean**	**SD**	**Mean**	**SD**	**Mean**	**SD**	**Mean**	**SD**	**Mean**	**SD**
30 min	4.1607	0.0015	4.2282	0.0077	4.2859	0.0142	4.3791	0.0200	4.6052	0.0000
20 min	4.2585	0.0074	4.4028	0.0201	4.4869	0.0448	4.4427	0.0351	4.6052	0.0000
10 min	4.3701	0.0019	4.4150	0.0093	4.5031	0.0329	4.5218	0.0465	4.6052	0.0000
5 min	4.4523	0.0063	4.4632	0.0047	4.4998	0.0164	4.5539	0.0186	4.6052	0.0000
0 min	4.5539	0.0000	4.6052	0.0023	4.6052	0.0000	4.6052	0.0000	4.6052	0.0000

Table S2. Optimized detection parameters and LC–MS/MS conditions

**Analytes**	**MRM transition(m/z)**	**DP (V)**	**CE (V)**	**LLOQ (ng/ml)**	**Retention time (min)**
Tacrolimus	821.5→768.6	25	30	1	3.75
Ascomycin	810.7→757.6	100	30	—	3.75
6β-hydroxyl testosterone	305.3→269.2	78	22	10	2.79
Prednisolone	361.3→343.2	22	15	—	2.79

Table S3 Parameters used for PBPK modeling of schisantherin A and schisandrin A

**Parameters**	**Schisantherin A**		**Schisandrin A**	
	**Value**	**Ref.**	**Value**	**Ref.**
Molecular weight (g/mol)	536.58	-	416.52	-
Compound type	neutral	-	neutral	-
Log $P_{o:w}$	1.7	In-house data	5.265	[1]
$f_{u\ plasma}$	0.083	[2]	0.0334	[2]
$f_{u\ gut}$	1	default value	1	default value
B/P	0.63	Predicted by Gastroplus®	0.69	Predicted by Gastroplus®
Intrinsic solubility (mg/mL)	0.01	Predicted by Gastroplus®	0.000668	Predicted by Gastroplus®
$P_{app,caco\text{-}2}$ ($\times10^{-6}$cm/s)	34.41	[3]		

PSA (Å^2)	-		55.38	https://pubchem.ncbi.nlm.nih.gov/
HBD	-		0	https://pubchem.ncbi.nlm.nih.gov/
K_p				
Adipose			36.4	
Brain			7.4	
Gut			210.3	
Heart	-	Predicted by Simcyp®	39.5	In-house data
Kidney			5.9	
Liver			3.9	
Lung			19	
Spleen			7.6	
$CL_{int,HLM}$(μL/min/mg protein)	24	In-house data	130	In-house data

Note: Log $P_{o:w}$, octanol/water partition coefficient; *f*u, fraction of unbound drug in plasma; B/P, blood to plasma partition ratio; $P_{app,caco-2}$,apparent permeability of Caco-2 cell line; PSA, polar surface area; HBD, hydrogen bond donors; K_p, tissue to plasma partition coefficients; $CL_{int,HLM}$, intrinsic clearance in human liver microsomes.

Table S4 Parameters used for PBPK modeling of tacrolimus

Parameter	Tacrolimus	
	**Value**	**Ref.**
Molecular weight (g/mol)	804.02	Drug label
Compound type	neutral	Drug label
Log$P_{o:w}$	3.26	[4]
$f_{u\ plasma}$	0.013	[4]
$f_{u\ gut}$	1	[5]
B/P	35	[4]
Intrinsic solubility (mg/mL)	0.012	[6]
$P_{app,caco-2,(pH\ 7.4:7.4),tacrolimus}$ (×10^{-6}cm/s)	13.1	[7]
$P_{app,caco-2,(pH\ 7.4:7.4)\ midazolam}$ (×10^{-6}cm/s)	32.4	[7]
CYP3A4 13-DMT *V*max	8 pmol/min/pmol rCYP3A4	[8]
CYP3A5 13-DMT *V*max	17 pmol/min/pmol rCYP3A5	[8]
CYP3A4 13-DMT *K*m, u	0.21 μM	[8]
CYP3A5 13-DMT *K*m, u	0.21 μM	[8]
CYP3A4 12-HT *V*max	0.6 pmol/min/pmol rCYP3A4	[8]
CYP3A5 12-HT *V*max	1.4 pmol/min/pmol rCYP3A5	[8]
CYP3A4 12-HT *K*m, u	0.29 μM	[8]
CYP3A5 12-HT *K*m, u	0.35 μM	[8]
CYP3A4/5 ISEF	0.24 (BD SUP)	Simcyp®
$CL_{R,\ human}$ (L/h^{-1})	0	[9]

Note: Log $P_{o:w}$, octanol/water partition coefficient; *f*u, fraction of unbound drug in plasma; B/P, blood to plasma partition ratio; $P_{app,caco-2}$, apparent permeability of Caco-2 cell line; 13-DMT, 13-O-desmethyl tacrolimus; 12-HT, 12-hydroxy tacrolimus; CL_R, renal clearance.

Table S5 C_{max} and AUC of tacrolimus under different K_p values

	CYP3A5 expresser		CYP3A5 non-expresser	
K_p	C_{max}[a] (ng/mL)	AUC[b] (ng/mL·h)	C_{max} (ng/mL)	AUC (ng/mL·h)
250	16.10 (1.14[c])	137.27 (2.25)	25.84 (1.06)	216.28 (1.82)
500	15.34 (1.09)	100.06 (1.64)	24.98 (1.03)	145.93 (1.23)
750	15.11 (1.07)	82.37 (1.35)	24.71 (1.02)	117. 92 (1.00)
1000	14..99 (1.06)	72.04 (1.18)	24.58 (1.01)	102.80 (1.16)
Observed	14.09	60.83	24.28	119.02

a Cmax: Maximal blood concentration; b AUC: Area under the curve; c fold error

Table S6 Demographic information used for PBPK modeling of tacrolimus

Characteristics	1 mg tacrolimus		2 mg tacrolimus		5 mg tacrolimus	
	CYP3A5 expressers	CYP3A5 non-expressers	CYP3A5 expressers	CYP3A5 non-expressers	CYP3A5 expressers	CYP3A5 non-expressers
Number (n)	12	26	31	40	12	12
Age (years)	23.0±1.0	23.0±3.3	23.0±1.7	23.0±1.8	24.0±1.4	21.0±0.5
Sex (male/female)	Male 100%		Male 100%		Male 100%	
Body weight (kg)	82.0±18.6	85.0±16.5	68.0±8.3	68.0±8.3	82.0±18.6	82.0±18.6
Height (cm)	179.0±8.1	178.0±7.8	174.0±6.4	174.0±6.7	179.0±8.1	179.0±8.1
Hematocrit (%)	44.0±2.7	43.0±3.0	46.0±4.0	46.0±3.8	44.0±2.7	44.0±2.7

Reference

1. Zhang M, Zheng J, Deng C, Song XM, Han L. Vinegar steam effect on oil - water partition coefficients of fructus schisandrae sphenantherae. Lishizhen Med Mater Med Res. 2012;23:2695-6.

2. Liang Y, Zhou YY, Liu YN, Guan TY, Zheng X, Dai C, et al. Study on the plasma protein binding rate of Schisandra lignans based on the LC-IT-TOF/MS technique with relative quantitative analysis. Chin J Nat Med. 2013;11:442-8.

3. Qin XL, Chen X, Zhong GP, Fan XM, Wang Y, Xue XP, et al. Effect of Tacrolimus on the pharmacokinetics of bioactive lignans of Wuzhi tablet (Schisandra sphenanthera extract) and the potential roles of CYP3A and P-gp. Phytomedicine. 2014;21:766-72.

4. Gertz M, Houston JB, Galetin A. Physiologically based pharmacokinetic modeling of intestinal first-pass metabolism of CYP3A substrates with high intestinal extraction. Drug Metab Dispos. 2011;39:1633-42.

5. Barter ZE, Perrett HF, Yeo KR, Allorge D, Lennard MS, Rostami-Hodjegan A. Determination of a quantitative relationship between hepatic CYP3A5*1/*3 and CYP3A4 expression for use in the prediction of metabolic clearance in virtual populations. Biopharm Drug Dispos. 2010;31:516-32.

6. Tamura S, Tokunaga Y, Ibuki R, Amidon GL, Sezaki H, Yamashita S. The site-specific transport and metabolism of tacrolimus in rat small intestine. J Pharmacol Exp Ther. 2003;306:310-6.

7. Gertz M, Harrison A, Houston JB, Galetin A. Prediction of human intestinal first-pass metabolism of 25 CYP3A substrates from in vitro clearance and permeability data. Drug Metab Dispos. 2010;38:1147-58.

8. Dai Y, Hebert MF, Isoherranen N, Davis CL, Marsh C, Shen DD, et al. Effect of CYP3A5 polymorphism on tacrolimus metabolic clearance in vitro. Drug Metab Dispos. 2006;34:836-47.

9. Bekersky I, Dressler D, Alak A, Boswell GW, Mekki QA. Comparative tacrolimus pharmacokinetics: normal versus mildly hepatically impaired subjects. J Clin Pharmacol. 2001;41:628-35
